# Modulation of Cellular Redox Status and Antioxidant Defense System after Synergistic Application of Zinc Oxide Nanoparticles and Salicylic Acid in Rice (*Oryza sativa*) Plant under Arsenic Stress

**DOI:** 10.3390/plants10112254

**Published:** 2021-10-22

**Authors:** Mohammad Faizan, Shafaque Sehar, Vishnu D. Rajput, Ahmad Faraz, Shadma Afzal, Tatiana Minkina, Svetlana Sushkova, Muhammad Faheem Adil, Fangyuan Yu, Abdulrahman A. Alatar, Firoz Akhter, Mohammad Faisal

**Affiliations:** 1 Collaborative Innovation Centre of Sustainable Forestry in Southern China, College of Forest Science, Nanjing Forestry University, Nanjing 210037, China; faizanetawah8@gmail.com (M.F.); fyyu@njfu.edu.cn (F.Y.); 2 Department of Agronomy, Institute of Crop Science, College of Agriculture and Biotechnology, Zhejiang University, Hangzhou 310058, China; 11816126@zju.edu.cn (S.S.); dradilfaheem@zju.edu.cn (M.F.A.); 3 Academy of Biology and Biotechnology, Southern Federal University, 344006 Rostov-on-Don, Russia; rajput.vishnu@gmail.com (V.D.R.); tminkina@mail.ru (T.M.); terra_rossa@mail.ru (S.S.); 4 School of Life Sciences, Glocal University, Saharanpur 247121, India; ahmadfaraz53@gmail.com; 5 Department of Biotechnology, Motilal Nehru National Institute of Technology Allahabad, Pryagraj 211004, India; shadmaafzalniazi11@gmail.com; 6 Department of Botany & Microbiology, College of Science, King Saud University, P.O. Box 2455, Riyadh 11451, Saudi Arabia; aalatar@ksu.edu.sa; 7 Department of Biomedical Engineering, Stony Brook University, New York, NY 11794-5281, USA; firozakhter86@gmail.com

**Keywords:** maximum quantum yield, reactive oxygen species, super oxide dismutase, X-ray diffraction

## Abstract

The objective of this research was to determine the effect of zinc oxide nanoparticles (ZnONPs) and/or salicylic acid (SA) under arsenic (As) stress on rice (*Oryza sativa*). ZnONPs are analyzed for various techniques viz., X-ray diffraction (XRD), Fourier-transform infrared spectroscopy (FTIR), transmission electron microscopy (TEM), and scanning electron microscopy (SEM). All of these tests established that ZnONPs are pure with no internal defects, and can be potentially used in plant applications. Hence, we further investigated for better understanding of the underlying mechanisms and the extent of ZnONPs and SA induced oxidative stress damages. More restricted plant growth, gas exchange indices, significant reduction in the SPAD index and maximum quantum yield (Fv/Fm) and brutal decline in protein content were noticed in As-applied plants. In contrast, foliar fertigation of ZnONPs and/or SA to As-stressed rice plants lessens the oxidative stress, as exposed by subordinate levels of reactive oxygen species (ROS) synthesis. Improved enzymatic activities of catalase (CAT), peroxidase (POX), and superoxide dismutase (SOD), proline and total soluble protein contents under ZnONPs and SA treatment plays an excellent role in the regulation of various transcriptional pathways participated in oxidative stress tolerance. Higher content of nitrogen (N; 13%), phosphorus (P; 10%), potassium (K; 13%), zinc (Zn; 68%), manganese (Mn; 14%), and iron (Fe; 19) in ZnONPs and SA treated plants under As-stress, thus hampered growth and photosynthetic efficiency of rice plants. Our findings suggest that toxicity of As was conquering by the application of ZnONPs and SA in rice plants.

## 1. Introduction

Exposure to arsenic (As) is a threat to humans, and the main source of exposure is water contaminated with As. Arsenic contaminated groundwater is reported in many parts of the world, and the most serious problems have been observed in different parts of India [1]. It exists in the environment both in inorganic [(Arsenate (AsV), arsenite (AsIII)] and organic forms. Arsenate is structurally similar to phosphate, so it is primarily ecstatic via elevated similarity phosphate transporters [2]. The presence of As in irrigation water and soil at increased levels could interfere with the normal growth and development of plants. Plants can develop symptoms of toxicity when exposed to excess As, either in the soil state or in solution cultures. It inhibits seed germination, root development, photosynthesis, and ultimately leads to plant death [3]. As an active redox metal, it also stimulates the generation of reactive oxygen species (ROS) that lead to lipid peroxidation, disturbance of cellular redox states and linked toxicity [4].

Various methods are used to overcome the adverse effects of abiotic stress on rice crops, including hybridization, genetic engineering, and molecular marker-assisted selective QTL mapping [5]. However, there are some errors in operating costs and technical expertise. In this scenario, a practical, inexpensive and feasible technology that can overcome these shortcomings is always in demand. Nanotechnology is an emerging field of the 21st century, and by introducing nanoparticles (NPs), nanorods, nanotubes, quantum dots, nanocarrier, nanodrug, nanosensors, etc., it is having a valuable impact on the global economy, industry and people’s lives [6]. Nanoparticles (at least less than 100 nm in one dimension) have unique optical properties that give them high quality surface-to-volume ratios and excellent physical, chemical and biological properties, depending on their size [7]. In agriculture, trace amounts of NPs are increasingly being evaluated as fertilizers to increase crop production under adverse conditions [5]. Therefore, the application of NPs is one of the new strategies to improve the plant growth and performance under trace metal stress. Evidence from both short-term and whole-crop life-cycle growth studies shows that trace amount of NPs could promote crop protection from abiotic stresses, although the results are likely due to ionic micronutrients as well [8].

Zinc, an essential micronutrient, plays an important role in many important metabolic processes. It may also help to increase chlorophyll and carotenoid biosynthesis and improved plant photosynthetic performance [9]. Zinc is involved in the action of a variety of enzymes (RNA and DNA polymerases, dehydrogenases, transphosphorylases, and proteinases), as well as the maintenance of the membranous structure and cell division, chlorophyll production, and the enhancement of the photosynthetic machinery of plants [10]. The ZnONPs have a large volume surface area ratio and have a significantly increased ultraviolet protection effect compared to bulk materials [7]. Exogenous application of ZnONPs significantly increased the seed germination, nitrogen absorption and seedling growth of rice [11]. Root and shoot length and leaf area of rice and wheat were also promoted in the presence of ZnONPs [12,13]. Dimpka et al. [8] reported that ZnONPs significantly enhanced the growth, yield, and uptake of macro and micronutrients of soybean plants under abiotic stress.

Salicylic acid is an essential signaling molecule and its role in protecting against various abiotic and biological stresses is well documented among plants [1]. The SA and acetylsalicylic acid (derivatives) have been used for therapeutic purposes for over a century. It is synthesized by two routes, the isocholysmate path and the phenylalanine ammonia-lyase pathway [14]. It binds to catalase (CAT), which initiates a cascade, to increase SA endogenous levels during abiotic and biological stress and increase hydrogen peroxide (H_2_O_2_) levels. As a result, enhanced H_2_O_2_ eventually recruits systemic acquired resistance and acts as a secondary messenger that induces the expression of pathogen-related proteins that increase plant defense [15]. SA has been reported to provide protection against trace metal stresses in various plants, including nickel in *Brassica juncea* and mercury in *Medicago sativa* [16,17]. Other studies have also shown protection through SA against Cd stress in soybean [18], barley [19] and rice [15] via modulation in antioxidant enzymes activity and photosynthetic performance.

Rice is a global stock with a population of 3 billion, with Asian countries accounting for 90% of production and consumption [20], and provides food security in many countries as a global food crop. Given these challenges, rice is still one of the top priority research crops. Arsenite (AsIII) is a major chemical species of As in paddy fields due to anaerobic cultivation conditions [21]. Additionally, most AsIII absorbed by plants is reduced to AsIII and preserved, so unlike other grains, rice brings together sulfhydryl groups of enzymes and proteins to make efficient AsIII that damages cell function. In rice, As is transported primarily via aquaporin channels and low silicon rice 1 and 2 (Lsi1 and Lsi2), which is an aquaporin like transmembrane protein and root-specific silicon carriers. Lsi1 and Lsi2 are confined to the exodermis of rice roots and distal and proximal to endothelial cells, respectively [22]. Rice grains contain 2–7% protein, including various essential and non-essential amino acids, such as glutamic acid (Glu), proline (Pro), and lysine (Lys). Lsi2 is reported to play an important role in transport of AsIII to shoots, and ultimately in the transport of rice grains. One study found a positive correlation between total amino acid content and As accumulation during trace metal stress [23]. It is reported that AsIII interferes with the function of proteins and enzymes through thiol interactions, while arsenates can displace phosphoric acid in many biochemical reactions that will lead to disruption of the flow of energy [4].

A combined application of ZnONPs and SA may have the potential to provide considerable tolerance against As-stress in rice plants. Keeping this in mind, we detect the effects of ZnONPs and SA on the oxidative damage and antioxidant enzymes activity transformation induced by As stress. At the field level, rice plant responses to combined application of ZnONPs and SA under As stress are remains unknown. Therefore, this research was planned to investigate the effective role of ZnONPs and SA for remediation of As stress in rice plants.

## 2. Results

### 2.1. Effect of As Toxicity on Rice Growth

**Figure 1:**
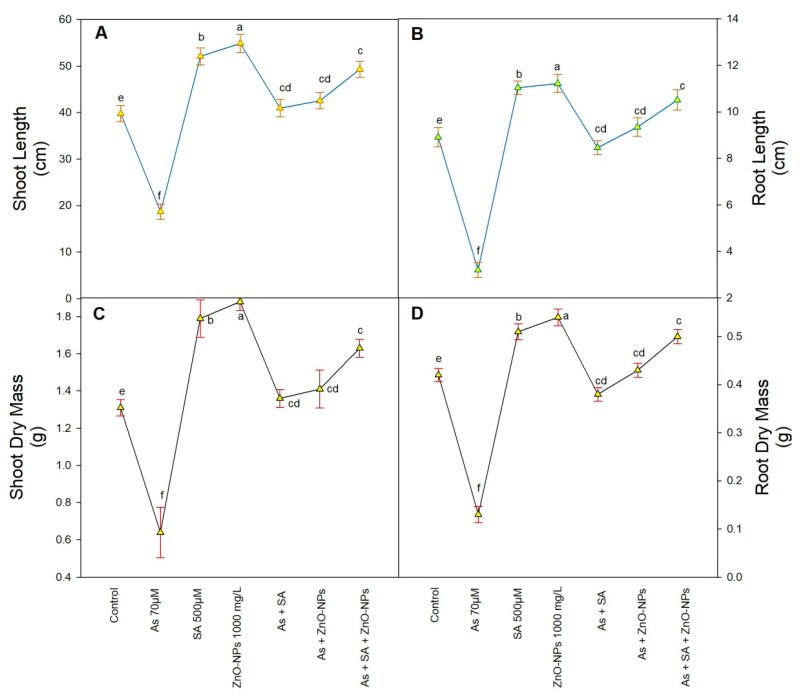
Effect of application of zinc oxide nanoparticles (ZnONPs), salicylic acid (SA) and their combination on growth of rice plants, shoot length (**A**), root length (**B**), shoot dry weight (**C**), and root dry weight (**D**) under arsenic (As) stress at the 50 day stage. All the data are the mean of 5 replicates (n = 5) and vertical bars demonstrated standard error (±SE). Different letters indicate significant differences among treatments at *p* < 0.05.

Shoot and root lengths, as well as their dry weights, were substantially reduced in As-contaminated plants when compared to control plants (Figure 1). In As contaminated plants, shoot length, root length, shoot dry weight and root dry weight reduced by 53%, 64%, 51%, and 67%, respectively, over their control plants. However, the foliar fertigation of ZnONPs and SA alone as well as in combination ameliorate the toxicity caused by As. In As containing plants, combined application of SA and ZnONPs increased the all aforesaid parameters by 24% (shoot length), 18% (root length), 25% (shoot dry weight), and 21% (root dry weight), respectively over non-treated plants (Figure 1).

### 2.2. Effect of SA and ZnONPs on Physiological Indices under As Stress

**Figure 2:**
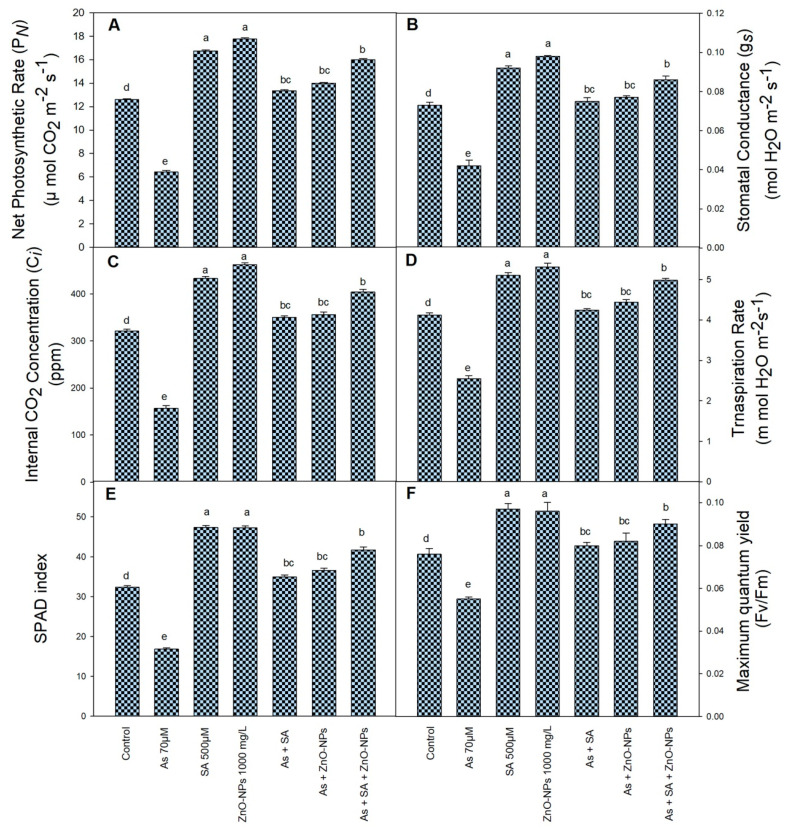
Effect of application of zinc oxide nanoparticles (ZnO NPs), salicylic acid (SA) and their combination on physio-chemical processes of rice plants, net photosynthetic rate (**A**), stomatal conductance (**B**), internal CO_2_ concentration (**C**), transpiration rate (**D**), SPAD index (**E**), and maximum quantum yield Fv/Fm (**F**) under arsenic (As) stress at the 50 day stage. All the data are the mean of 5 replicates (n = 5) and vertical bars demonstrated standard error (±SE). Different letters indicate significant differences among treatments at *p* < 0.05.

Arsenic stress significantly reduced the activity of gas exchange characteristics such as net photosynthetic rate (P_N_), stomatal conductance (gs), internal CO_2_ concentration (Ci), and transpiration rate (E) by 49%, 2%, 51%, and 38%, respectively, over to water treated plants (Figure 2A–D). However, fertigation of ZnONPs and SA partially or completely diminished the toxicity caused by As stress. Moreover, combined application of SA and ZnONPs in the presence of As increased the P_N_ by 27%, gs by 19%, Ci by 26%, and E by 21%, respectively, over water treated plants (Figure 2A–D). The SPAD index and Fv/Fm also followed the same pattern in the presence of As, SA and ZnONPs and increased the SPAD by 29% and Fv/Fm by 19% over their respective control (Figure 2E,F).

### 2.3. Combined Application of SA and ZnONPs on Antioxidant Enzyme Activity under As Stress

**Figure 3:**
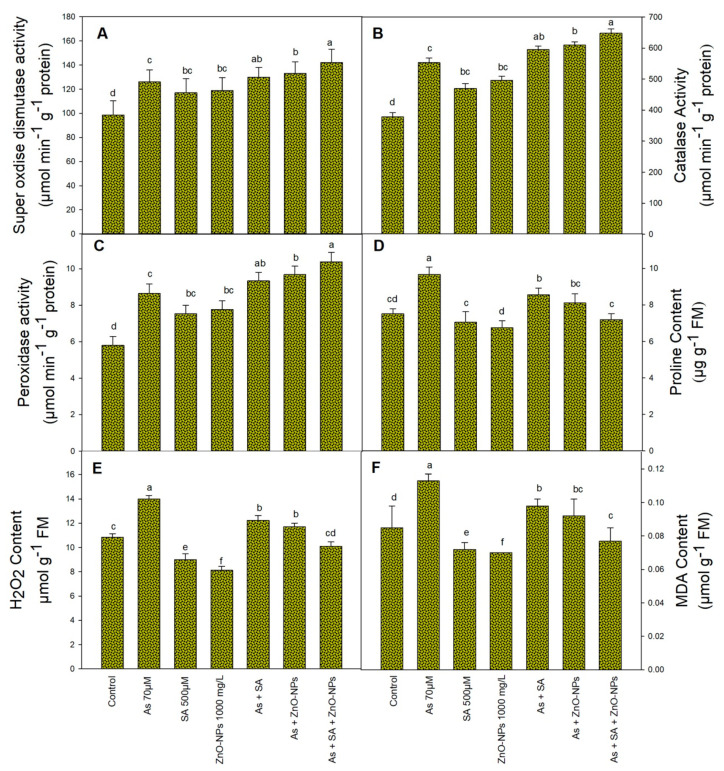
Effect of application of zinc oxide nanoparticles (ZnONPs), salicylic acid (SA) and their combination on antioxidant enzymes activity of rice plants, super oxide dismutase (**A**), catalase (**B**), peroxidase (**C**), proline (**D**), hydrogen peroxide (H_2_O_2_) (**E**), and malondialdehyde (MDA) (**F**) under arsenic (As) stress at the 50 day stage. All the data are the mean of 5 replicates (n = 5) and vertical bars demonstrated standard error (±SE). Different letters indicate significant differences among treatments at *p* < 0.05.

Arsenic stress significantly improved the activities of CAT (46%), POX (49%), and SOD (28%) in comparison to non-treated plants (Figure 3A–C). Following-up treatment with ZnONPs and SA, either alone or in combination, resulted in an increase in the activities of the aforesaid enzymes. The maximum enhancement of CAT (71%), POX (79%), and SOD (44%) was recorded in the plants treated with ZnONPs and SA in the presence of As (Figure 3A–C).

### 2.4. Effect of SA and ZnONPs As a Foliar Fertigation on H_2_O_2_, MDA and Proline Content under As

Arsenic stress considerably affected the lipid peroxidation and increased MDA level, while SA and ZnONPs treated plants considerably reduced MDA content formation (Figure 3F). MDA content increased by 33% in only As-treated plants, however, combined application of SA and ZnONPs significantly reduced MDA content by 47% in comparison to As-treated plants (Figure 3F). Under As stress, H_2_O_2_ content was significantly increased (29%), but the supplementation of SA and ZnONPs reduced the H_2_O_2_ content alone as well as in combination (Figure 3E).

Proline content considerably enhanced in the As-treated plants and the increment was 29%, compared to control (Figure 3D). However, foliar fertigation of SA and ZnONPs reduced the proline content alone as well as in combination. The reduction in proline content after exogenous treatment of SA and ZnONPs by 34% as compared to only As treated plants (Figure 3D).

### 2.5. Total Soluble Protein and As Content

**Figure 4:**
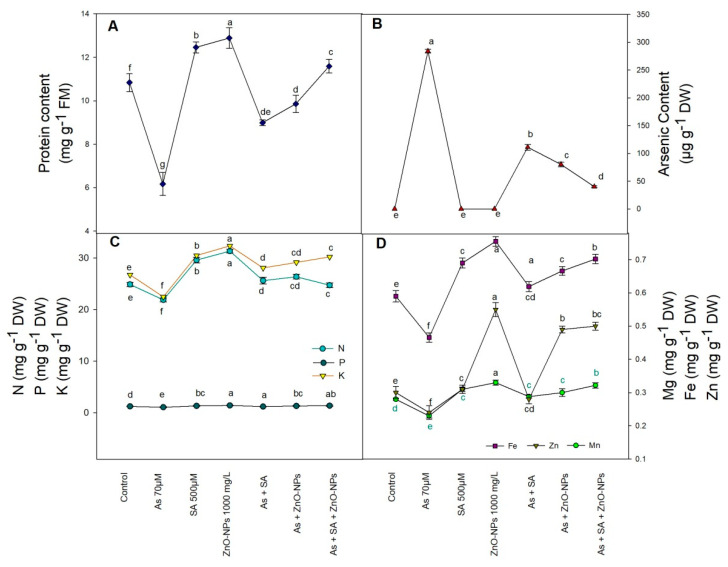
Effect of application of zinc oxide nanoparticles (ZnONPs), salicylic acid (SA) and their combination on elemental status of rice plants, protein content (**A**), As amount (**B**), nitrogen, phosphorus, potassium (N, P, K) (**C**), and iron, zinc, manganese (Fe, Zn, Mn) (**D**) under arsenic (As) stress at the 50 day stage. All the data are the mean of 5 replicates (n = 5) and vertical bars demonstrated standard error (±SE). Different letters indicate significant differences among treatments at *p* < 0.05.

Protein content was significantly reduced by the amendment of As, the reduction was 49% over the control one (Figure 4A). However, negative effects caused by As completely and partially overcome by the application of SA and ZnONPs. Combined application of SA and ZnONPs completely neutralized the adverse effects of As and increased the protein content by 88% in comparison to only As-treated plants (Figure 4A).

Rice plants grown in As-contaminated soil showed augmented accretion of As amount in roots over control (Figure 4B). Foliar fertigation of SA and ZnONPs resulted in reduced content of As in roots by 86% compared to As-treated plants (Figure 4B).

### 2.6. Arsenic Mediated Accumulation in Different Elements

Macro and micro nutrients (N, K, Zn, Mn, and Fe) of rice leaf tissues were considerably reduced under As-stress by 12% (N), 16% (K), 18% (Zn), 15% (Mn), and 21% (Fe), respectively, over control plants (Figure 4C,D). Compared to water treated plants, spraying ZnONPs and SA alone as well as in combination increased their leaf tissue content of micro and macro nutrients. Among all treatments, plants subjected with SA and ZnONPs under As-stress increased the content of N, K, Zn, Mn, and Fe by 13, 34, 108, 40, and 51%, respectively, over only As-treated plants.

## 3. Discussion

Studies reveals that NPs have ability to mitigate the stress caused by trace metal and enhanced the growth of the plants [24,25]. Despite several studies of NPs with plants, the mechanism of ZnONPs and SA in the alleviation of oxidative stress induced by As in rice plant is not clearly developed.

Various scientists have accounted the undesirable effects of As-toxicity on plant growth and development. It is obvious that As encourages adverse impacts on plants and obstructs growth, photosynthesis, and protein accumulation in plants [26,27]. Rice is the highly pretentious staple crop to As-stress over with *Triticum aestivum*, *Zea mays*, and *Hordeum vulgare* because of its farming in flooded situation as compared to non-flooded for *Triticum aestivum* [28]. In the present study, growth (length of shoots and root, dry weight of shoot and root) was significantly decreased in plants receiving 70 µM of As due to the formation of higher oxidative stress (Figure 1A–D). Several studies have already shown the adverse effects of As on plants [27]. There are some findings showing negative effects of As on the growth of *Vicia faba* [26], *Arundo donax* [29], and *Linum usitatissimum* [30].

While in the presence of As, rice seedlings treated with ZnONPs and SA showed significant increases in shoot length, root length, shoot dry weight, and root dry weight (Figure 1A–D). Plant growth in terms of length and biomass is considered as one of the reliable indicators of abiotic stress [31]. Previously, Ahmad et al. [32] reported that application of ZnONPs significantly increased the plant height, dry weight, and photosynthetic rate of *Glycine max* under As stress. Ullah et al. [33] demonstrated that the treatment of adequate Zn significantly increased the length, fresh and dry weight, and area of leaves of *Cicer arietinum* under stress conditions. Furthermore, Yan et al. [34] reported that the growth indices and photosynthesis were considerably reduced in rice plants under As stress; however the foliar application of ZnONPs appreciably increased plant height and chlorophyll content compared with control plants. Correspondingly, it’s described that ZnONPs application increased plant height, weight of shoot and root (fresh as well as dry) and leaf area of *Solanum lycopersicum* under Cu and salt stress, respectively [35]. Salicylic acid play important role as a primary signaling molecule and it is concerned in lessening of stress [1]. Kaya et al. [36] record that the As-stresses significantly decreased the growth; while the foliar application of SA increased the growth of the plant under As-stress. Salicylic acid is involved in the regulation of plant metabolism in stress environment and thus offers shield in plants against abiotic stresses [37]. Application of SA to stressed plants was accounted to trigger key abiotic stress tolerance mechanisms [38].

Higher photosynthetic rates can assurance an elevated grain yield. In the whole productivity of crops, only 5–10% of the nutrients are from the soil to roots, and 90–95% is from photosynthesis [39,40]. Photosynthesis in advanced plants is very susceptible to metals restraining the chlorophyll biosynthesis [41]. Arsenic toxicity decreased photosynthesis and related attributes; chlorophyll content and maximum quantum yield (Fv/Fm) in rice and *Ceratophyllum demersum* [42]. However, SA and ZnONPs application increased photosynthesis related attributes, SPAD index and Fv/Fm under As stress in rice plants (Figure 2A-D), as previously observed in *Triticum aestivum* [43] and *Glycine max* [32]. Elevated performance of P_N_ and related attributes and reduced H_2_O_2_ content is related to the SA and ZnONPs appliance, recommend that SA contributed in alleviating the injurious consequences of As on P_N_ and related attributes, most likely by dropping the formation of H_2_O_2_ as previous reported in *Artemisia annua* L. [44]. It’s understood that Zn plays an important function in photosynthesis, encouraging the augmentation of chlorophyll amount. Current researchers found that the application of ZnONPs augmented the chlorophyll content of plants under Cd and Pb stress [45]. The amelioration of photosynthesis by the application of SA to As-treated plants can be due to the improvement in the activity of ribulose-1, 5-bisphosphate carboxylase oxygenase (RuBisCO) enzyme as suggested by Ahmad et al. [46]. Our results are lined with the earlier studies of Faraz et al. [47] to improving the photosynthetic related attributes by the application of SA under Cd stress in *Brassica juncea*.

To subsist with As-triggered oxidative stress, plants have developed ROS-scavenging enzymatic and non-enzymatic antioxidants [48]. Arsenic stress increased the antioxidant activities in *Trigonella goenum-graecum* [49], *Zea mays* [50], and *Ocimum tenuiflorum* [51]. In our study, the exogenously applied ZnONPs and SA significantly increased the antioxidant enzymes activity (Figure 3A–C) as they developed the antioxidative mechanism, indicated that ZnONPs and SA perhaps elevate the level of stress-responsive gene expression and ROS accumulation, which recovers the development under stressful conditions [36]. Foliar fertigation of ZnONPs considerably improved SOD and CAT activities, as it is an important component that can induce gene expression of antioxidant enzymes under NaCl stress [52]. Activity of antioxidant enzymes was significantly increased by the introduction of As stress alone; however, it was further increased in rice seedlings exposed with ZnONPs and SA (Figure 3A–C). Due to the high adsorption capability of ZnONPs, As is adsorbed previous to it enters the rice plant, thus defensive the antioxidant mechanism of rice [53]. It has been observed that treatment of ZnONPs de-escalate the generation of ROS in the presence of Cd stress in tomato [54]. Similar to this, studied by Ahmad et al. [32] in soybean and Shemi et al. [55] in maize also reported the same results. Recent study revealed that ZnONPs and SA shows helpful result on antioxidant enzymes actions in *Brassica napus* [56] and *Lippia citriodora* [57]. Cumulatively, present is an augmented intensity of activity of antioxidant enzymes documentation to rice plants treated with ZnONPs and SA under As stress. This designated the constructive role of ZnONPs and SA in the alleviation of As induced oxidative damage.

Increased formation of ROS under As stress exposure basically consequences in oxidative damage that hampered lipid peroxidation of membranes basis disturbance of their integrity, i.e., MDA increased [4]. In the current study, oxidative stress is indicated by increased amount of H_2_O_2_ and MDA in plant treated with As (Figure 3E,F). It is lined with the study of Tripathi et al. [58]. Fluctuation in redox homeostasis after As application considered as the primary factor for hindered growth in rice plants [59]. ZnONPs and SA application diminishes toxic effects of As raised by H_2_O_2_ and MDA, which demonstrated that ZnONPs and SA protect the crop against oxidative stress caused by As. In line with this, a similar protective role of SA has been shown under Cd stress in barley [19] and against glyphosate-based herbicide stress in tomato [60].

Proline play important job in osmoregulation in stress circumstances [61]. It lowers the latent osmotic cell’s capacity to provide high turgor competence, which increases the plant’s ability to optimize storage reserves to sustain the metabolism of the process under stressful circumstances while also maintaining the plant’s growth and development. The accumulation of proline is caused by an increase in protein degradation and conversion to amino acids, which includes proline, or by an increase in the activity of the proline biosynthesis enzyme, or through a decrease in proline oxidation [62]. ZnONPs and SA resulted in a reduction in proline accumulation in the As-treated plants as compared to the control plants (Figure 3D). Our obtained results are in the conformity with the study of Lutts et al. [63] and Faizan et al. [64].

ZnONPs and SA exhibited a controlling impact on rice As uptake. The amount of As significantly decreased by the application of SA and ZnONPs, indicating that SA and ZnONPs may serve as an As barrier to limit the As uptake by plants. In addition, since the plasma membrane is selectively permeable, it may regulate the transport and exchange of materials both inside and outside the cell. Results shown in Figure 4B revealed that ZnONPs had the ability to absorb As and decrease the amount of As present in rice plants. As a result of the disruption of the root cell membrane’s integrity, the application of ZnONPs has been shown to exacerbate the degradation of the root cell membrane. Similarly, Lata and Samadder, [65] and Singh et al. [53] reported that ZnONPs were used for removal of As in water. It is found that CuONPs reduced As uptake in rice by deleting As from the solution with the precipitated complexes [66]. Our results are corroborated by the previous studies of Conway et al. [67] and Sharifan et al. [68].

To know the plant physiological status, protein content may prove an impressive indicator [69]. In the current observation, the level of protein drastically reduced in As-treated rice plants (Figure 4A). However, when ZnONPs and SA are exogenously applied, As treated plants exhibited a gradual surge in protein content in comparison to non-treated as well as As-treated plants. This result showed that As treatment induces metabolic disorders in the rice plants, and application of ZnONPs and SA may counterattack the response of As treatments [70]. In addition to this, Faizan et al. [54], in tomato plant in the presence of Cd, reported the similar results.

Arsenic stress considerably decreased the amount of macronutrients (N, P, and K) and micronutrients (Zn, Mn, and Fe) in rice plant leaves (Figure 4C,D). The reason behind the reduction of these nutrients in the presence of As is that the high ion amount in the soil that exaggerated on the commencement of the enzyme within the plant cells repressed the active sites of enzymes, and due to metabolism disorder by H+-ATPase pumps break down [71]. However, application of SA and ZnONPs significantly increased the amount of nutrients in plant leaves, because of the induction of the antioxidant enzymes system by increased activity of CAT, POX, and SOD and reduced the oxidation of photosynthesis pigments [72]. The present results are in line with the study of Lamhamdi et al. [73] on wheat and spinach.

## 4. Materials and Methods

### 4.1. Characterization of Zinc Oxide Nanoparticles

**Figure 5:**
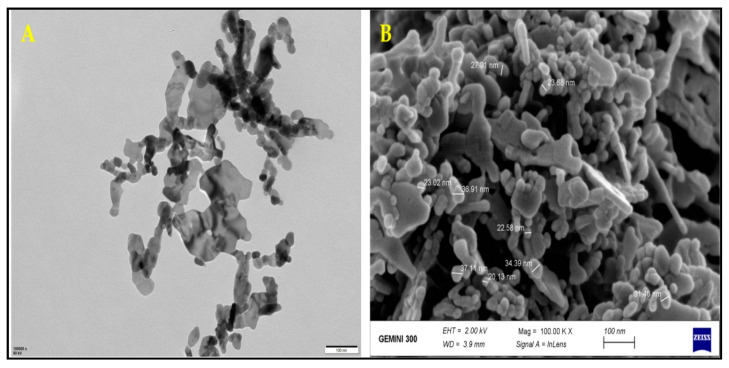
Characterization of ZnONPs, (**A**) TEM: transmission electron microscopy of ZnONPs, and (**B**) SEM: scanning electronic microscopy.

**Figure 6:**
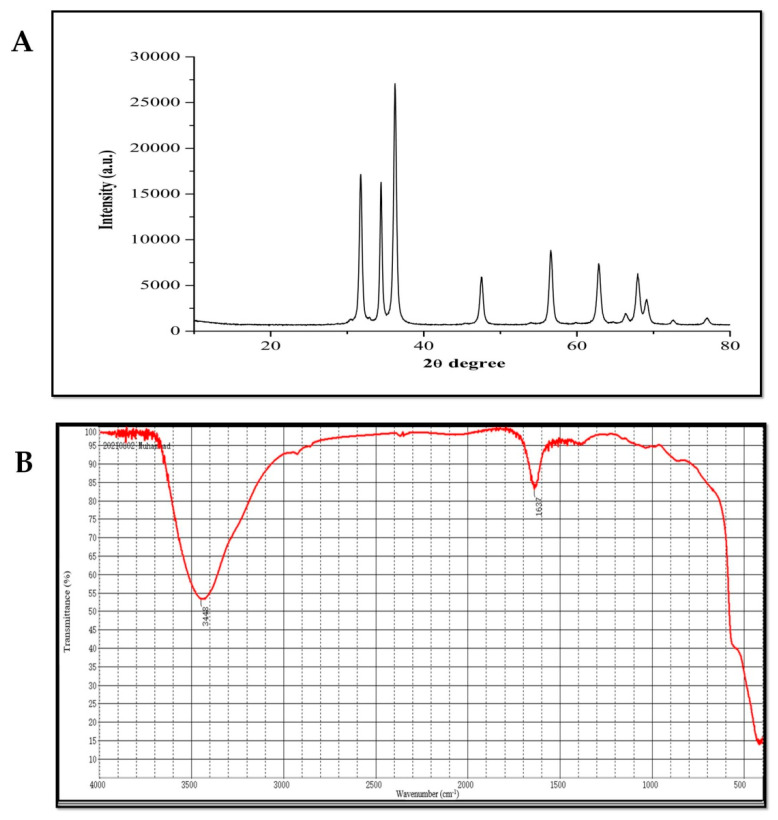
Typical XRD pattern of ZnONPs (**A**) and typical FTIR spectra of ZnONPs (**B**).

Zinc oxide NPs (purity 99.9%) were purchased from Cw-nano (www.cwnano.com; accessed on 23 January 2021). ZnONPs were ready in double distilled water, and the suspension was sonicated in an ultrasonic bath (Shanghai Kudos Ultrasonic Instrument Co., Ltd., Shanghai, China) for 30 min previous to examination. Morphological and structural characterization of ZnONPs was performed by TEM, SEM, XRD and FTIR analyses as shown in Figure 5 and Figure 6. Surface morphology and particle size of ZnONPs were determined via TEM (JEM-1230, JEOL, Akishima, Japan) and SEM (TM-1000, Hitachi, Japan), however, TEM image concluded that the ZnONPs have a spheroidal shape with an average size of 30 nm and length 0.2 µm (Figure 5A). Furthermore, SEM image showed the uniform distribution of the particles (Figure 5B). For SEM analysis, the NPs was fixed on an aluminum stub using carbon double-sided glue tabs and enclosed with thin conductive palladium film, while a 200-mesh carbon-coated copper grid was used for TEM analysis. Furthermore, to analyze the crystallite structure of ZnONPs, XRD analysis was performed according to Nabila and Kannabiran [74] and the data were collected with 2θ ranging from 20° to 80°. The diffraction peaks at (100), (002), (101), (102), (110), (103) and (112) revealed in the XRD pattern of the pure ZnONPs planes clearly show their crystalline nature are shown (Figure 6A). The particle size was measured by Scherrer’s equation (d = Kλ/β cos θ) from the XRD pattern and the average particle size was calculated to be 20 nm with little dispersion in size. The presence of surface coating functional groups of NPs was confirmed through FTIR (Spectrometer vector 22, Bruker, Germany) in the spectral range of 4000–500 cm^−1^ at room temperature [75]. FTIR analysis showed multiple spectral peaks at 3448 and 1637 cm^−1^. Overall, FTIR spectra revealed that various functional groups from diverse classes of molecules are involved in the stabilization of ZnONPs (Figure 6B).

### 4.2. Plant Material, Salicylic Acid and Zinc Oxide Nanoparticle Treatments and Sample Collection

Rice seeds (*Oryza sativa;* var. VL Dhan 221) were used in the present study. Seeds were surface sterilized with the help of 70% (*v*/*v*) of ethanol up to 1 min and then 30% (*v*/*v*) of sodium hypochlorite next for 8 min. After that, seeds were washed thoroughly using double distilled water (DDW). Germinated seeds were transferred to the maintained pots (35 in number). The 14-days-old seedlings were treated with 70 µM of As (sodium arsenite, NaAsO_2_) via soil. SA (500 µM) and ZnONPs (1000 mg/L) were foliarly applied for seven days starting from 35 days after sowing. The experiment was executed in a randomized complete block design with a factorial arrangement of five replicates per treatment. The growth indices as well as biochemical and physiological attributes of rice plants have been assessed at 50 DAS.

### 4.3. Morphological Parameters

Plants were harvested after 50 DAS, uprooted with attached soil, washed with water to removed soil particles and cut into shoot and root. Their length was measured using a meter scale. After that, root and shoot were kept in an oven (70 °C) for 72 h to dry the samples, after which dry weights were measured.

### 4.4. Physiological Measurement

P_N_ (µmol CO_2_ m^−2^ s^−1^), gs (mol H_2_O m^−2^ s^−1^), Ci (ppm), and E (m mol H_2_O m^−2^ s^−1^) were measured with Portable Infra-Red Gas Analyzer (LI-COR 6400, LI-COR Biosciences, Lincoln, NE, USA) in full sunshine (11:00–12:00). For measuring P_N_, E, gs, and Ci, the method adopted is as described by Faizan et al. [76].

On the same day, the SPAD index was determined using SPAD (Soil Plant Analysis Development) chlorophyll meter (SPAD-502; Konica, Minolta Sensing, Inc., Sakai, Osaka, Japan).

For quantum yield (Fv/Fm) measurement, mature leaves of rice plants were selected and store in dark for about 30 min by using the leaf chamber fluorometer (LI-COR 6400-40, LI-COR Biosciences, Lincoln, NE, USA). For determination of Fv/Fm method adopted as described by Faizan et al. [77].

### 4.5. Antioxidant Enzymes Analysis

The activities of CAT, POX, and SOD were determined by method followed by Faizan and Hayat [78]. For the determination of CAT, POX, and SOD, the leaf (0.5 g) was homogenized in a 50 mM phosphate buffer (pH = 7) of 1% polyvinylpyrrolidone. These mixtures were centrifuged at 15,000× *g* for 10 min at 4 °C, and the final supernatant was used as a source for the determination of CAT, POX and SOD. For determination of POX activity, the enzyme extract (0.1 mL) was mixed in the reaction mixture of pyrogallol, phosphate buffer (pH = 6.8) and 1% H_2_O_2_. The absorbance was measured at 420 nm on a spectrophotometer. The estimation of CAT, a mixture was prepared containing a phosphate buffer (pH = 6.8), 0.1 M H_2_O_2_ and enzyme extract (0.1 mL). H_2_SO_4_ was mixed in the reaction mixture after its incubation for 1 min at 25 °C, and was titrated against a potassium permanganate solution. For SOD, the preparation of the reaction mixture, 50 mM phosphate buffer (pH = 7.8), 20 µM riboflavin, 75 mM NBT, 13 mM methionine and 0.1 mM ethylene diamine tetra acetic acid (EDTA) were required. The mixture was irradiated within two fluorescent light tubes for 10 min and absorbance was noted at 560 nm using a UV–visible spectrophotometer.

### 4.6. Determination of Proline, Hydrogen Peroxide, Malondialdehyde and Total Soluble Protein

The method of Bates et al. [79] was used for the purpose of proline content determination in plant leaves. For estimation, 50 mg of leaves was extracted in sulfosalicylic acid, and same amount of glacial acetic acid and ninhydrin solutions were also mixed. Sample was heated at 100 °C, to which 5 mL of toluene was added. Absorbance of the aspired layer was noted at 528 nm on a spectrophotometer.

For lipid peroxidation analysis, MDA content was measured by following the method of Heath and Packer [80]. The concentration of H_2_O_2_ in the leaves was measured as Patterson et al. [81] protocol. Leaf tissues (0.5 g) were homogenized in 10 mL cold acetone using mortar and pestle. The homogenate was centrifuged at 5000× *g* for 15 min and the supernatant was kept. Residue was again extracted with acetone. Then, 1 mL of the supernatant was taken in the test tube and 2 mL of 17 M ammonia and 2 mL of 20% titanium chloride (prepared in conc. HCl) were added. The supernatant was again extracted with acetone, accompanied by an infusion of 10 mL 2 N H_2_SO_4_ to absorb it properly. The reaction mixture was again centrifuged to remove the undissolved inputs. The optical density was measured at 410 nm on a spectrophotometer against blank. Content of H_2_SO_4_ in the plant samples was assessed in relation to the standard curve adopted from the known concentration of H_2_O_2_ and was formulated as μ mole g^−1^ FM.

Bradford’s method [82] was used to determine the amount of protein in the sample. The process of Faizan et al. [83] was repeated to evaluation of protein content. For this 1 g of newly formed leaves were homogenized in buffer consisted of 40 mM tris-HCl (pH = 7.5), 0.07% β-mercaptoethanol, 2% polyvinylpyrrolidone, 0.5% Triton X-100, 1 mM phenyl methane sulfonyl floride (PMSF) and 1 mM EDTA by pestle and mortar, and mixture was centrifuged at 20,000× *g* for 10 min. The supernatant was collected, and Bradford reagent was added for color development. Intensity of color was read at spectrophotometer, and protein content was expressed as mg g^−1^ (FW).

### 4.7. Accumulatin Analysis of Different Elements

The estimation of nutrients (N, P, K, Fe, Mn, and Zn) was measured in dry, fine powder of rice leaves. A micro-Kjeldahl (Medic. Instr. Co., Ningbo, China) apparatus was used to measured N content [84]. For measurement of P, the Chapman and Pratt [85] procedure was followed. However, an Atomic Absorption Spectrophotometer (Model 3300, PerkinElmer, Waltham, MA, USA) was used to evaluate the amount of Zn, Mn, and Fe in plant leaves as described by Nassar and El-Sahhar [86].

### 4.8. Estimation of As

Content of As in roots was determined in samples dried up to 48 h at 80 °C. The prepared fine powder of the samples was digested with HNO_2_/HClO_4_ (3:1, *v*/*v*) concentrated mixture. Arsenic content was determined in samples by Atomic Absorption Spectrophotometer (GBC, 92 plus, GBC Scientific Instruments, Braeside, Australia).

### 4.9. Statistical Analysis

Statistical study of the data was carried out with the help of SPSS ver. 17 (IBM Corporation, 1 New Orchard Road, Armonk, New York, NY, USA). LSD was deliberated with ANOVA, and Duncan’s multiple range test was used to differentiate means.

## 5. Conclusions

This study highlights that the foliar fertigation of ZnONPs in the presence of SA ameliorates the As-induced oxidative stress in rice plants by instantaneous prompting of the mechanism of the antioxidant system. Further, examinations such as large-scale field checks could be helpful in raising a sustainable crop production system, particularly in As-infected areas. Additionally, the report information unlocks novel frontiers of fundamental study in the crosstalk between ZnONPs and SA, which should allow decoding at the molecular rank.

## Data Availability

Not applicable.
